# Genistein induces degradation of mutant huntingtin in fibroblasts from Huntington’s disease patients

**DOI:** 10.1007/s11011-019-00405-4

**Published:** 2019-03-09

**Authors:** Karolina Pierzynowska, Lidia Gaffke, Zuzanna Cyske, Grzegorz Węgrzyn

**Affiliations:** Department of Molecular Biology, University of Gdańsk, Wita Stwosza 59, 80-308 Gdańsk, Poland

**Keywords:** Huntington’s disease, Genistein, Huntingtin, Protein degradation, Protein aggregates, Autophagy

## Abstract

Mutations in the *HTT* gene, consisting of expansion of CAG triplets, cause the Huntington’s disease (HD), one of the major neurodegenerative disorders. Formation of aggregates of mutant huntingtin (mHTT, the product of the mutant *HTT* gene) leads to cellular dysfunctions, and subsequent neurodegeneration which manifest clinically as motor abnormalities and cognitive deficits. We recently used immortalized HEK-293 cells expressing the 1st exon of the mutant *HTT* gene as a cellular model of HD, and showed that the stimulation of autophagy by genistein corrected the mutant phenotype. However, effects of genistein on HD patient-derived cells remained unknown. In this report, we demonstrated that genistein also instigated degradation of mHTT in fibroblasts derived from HD patients. This was assessed as a significant decrease in the levels of HTT in HD fibroblasts measured by Western-blotting, and the disappearance of intracellular mHTT aggregates in cells observed by fluorescent microscopy. Fibroblasts derived from control persons were not affected by genistein treatment. These results indicate that genistein can improve HD phenotype in patient-derived cells, and substantiates the need for further studies of this isoflavone as a potential therapeutic agent.

## Introduction

Huntington’s disease (HD) is an autosomal dominant genetic disorder which is manifested clinically by specific symptoms including chorea, psychiatric disturbances and cognitive decline (Morreale 2015). This neurodegenerative disease is fatal, as no effective treatments are currently available (Shannon and Fraint 2015).

HD is caused by mutations in the *HTT* gene, coding for huntingtin (HTT), which result in the expansion of CAG repeats (Morreale 2015). This expansion results in the formation of a long tract of glutamine residues (the polyQ tract) in the mutant huntingtin protein (mHTT). Due to misfolding of polyQ-containing domains, mHTT forms aggregates that cause cellular dysfunctions resultant in progressive neurodegeneration (Morreale 2015). It is generally accepted that 40 or more CAG repeats in the 1st exon of *HTT* lead to full penetrance of the mutation and the occurrence of HD-specific symptoms (as stated by American College of Medical Genetics and the American Society of Human Genetics).

Although no effective treatments of HD are available, various approaches to improve cell viability by inhibiting expression of the mutant *HTT* allele, the inactivation of mHTT, and/or the degradation of mHTT were tested (Shannon and Fraint 2015). It has been suggested recently that stimulation of the autophagy process might be the most promising strategy for development of effective therapies for diseases caused by accumulation of pathological macromolecules, including mHTT (Guo et al. 2018; Pierzynowska et al. 2018a). However, the vast majority of autophagy stimulators characterized to date do not meet the criteria to be used as long-term therapeutics crucial for the treatment of neurodegenerative diseases (Yang et al. 2013). Particularly, dose- and/or duration-dependent overactivation of autophagy might potentially cause severe adverse effects. Genistein (trihydroxyisoflavone or 5, 7-dihydroxy-3- (4-hydroxyphenyl)-4*H*-1-benzopyran-4-one), a potent autophagy stimulator, might be a putative drug for the treatment of neurodegenerative diseases, because it crosses the blood-brain-barrier, and is safe when administered even at high doses (like 150 mg/kg/day) for several months to animals and humans (Malinowska et al. 2009, 2010; Kim et al. 2013; Moskot et al. 2014). Our recent studies, using immortalized HEK-293 cells transiently transfected with a plasmid expressing the 1st exon of the *mHTT* gene as a cellular model of HD showed that genistein-mediated induction of autophagy corrected the HD phenotype (Pierzynowska et al. 2018b). Although these results are promising, a demonstration of beneficial effects of genistein in HD patient’s cells is necessary.

Although HD primarily affects the brain, *HTT* is ubiquitously expressed also in peripheral cells (Sharp et al. 1995; Kegel et al. 2002). Moreover, HD fibroblasts manifest cellular dysfunctions similar to neural HD cells (Sassone et al. 2009; Marchina et al. 2014; Petersen et al. 2014; Jędrak et al. 2017a). Since fibroblasts can be readily obtained from patients, and they can be easily cultured under laboratory conditions, we decided to assess the efficacy of genistein in HD patient-derived fibroblasts.

## Materials and methods

### Formal and ethical issues

This study was conducted according to the tenets of the Helsinki Declaration, and it was approved by the local Ethics Committee of the Medical University of Gdansk (NKEBN/254/2011 and NKEBN/254–431/2012). Written informed consents were obtained from all donors of the biological material, both HD patients and persons from the control group, prior to the study procedures.

### Human subjects

Biological material from four HD patients and four age- and sex-matched controls was used in this study (information about the written informed consents is provided in the previous subsection). The Unified Huntington’s Disease Rating Scale (UHDRS) was used to assess HD severity (Huntington Study Group 1996). Psychiatric features were assessed by the Clinical Global Impressions scale (CGI), and the functional assessment was measured by the Total Functional Capacity scale (TFC), as described previously (Jędrak et al. 2018). Characteristics of the HD patient and control groups is presented in Table 1.

**Table 1:** Characteristics of patients and control subjects at the time of skin biopsy

Subject	Sex	Age (y)	No. of CAG repeats	Rating scale		Duration of motor symptoms (y)
				CGI	TFC	
Control 1	M	51	NT	NA	NA	NA
Control 2	M	50	NT	NA	NA	NA
Control 3	M	51	NT	NA	NA	NA
Control 4	M	43	NT	NA	NA	NA
HD 1	M	41	43	3	II	17
HD 2	F	45	43	4	II	4
HD 3	M	54	43	5	III	13
HD 4	M	49	42	4	I	2

Abbreviations: CGI, Clinical Global Impressions scale; TFC, Total Functional Capacity scale; M, male; F, female; NT, not tested; NA, not applicable

### Cell cultures

Biopsies were taken from the forearm skin, and fibroblast lines were established as described previously (Jędrak et al. 2017a, 2017b, 2018). Fibroblasts were cultured on 10-cm plates in DMEM (Thermo Fisher Scientific Inc., Paisley, UK) supplemented with 10% FBS (Thermo Fisher Scientific Inc., Paisley, UK) and 1% antibiotic/antimycotic solution (Sigma-Aldrich Co. LLC., St. Louis, USA) at 37 °C under a humidified atmosphere of 95% air/5% CO_2_.

### Reagents

Genistein (99% purity; #446–72-0) was purchased from Pharmaceutical Research Institute, Warsaw, Poland). It was dissolved in DMSO at stock concentrations of 30, 60, and 100 mM, and stored at -20 °C. The following antibodies were used: monoclonal mouse anti-human huntingtin clone mEM48 (#MAB5374 for immunofluorescence), and clone 1HU-4C8 (#MAB2166 for Western-blotting) (Sigma-Aldrich, city, Munich, Germany); goat anti-mouse conjugated with Alexa Fluor 488 (#A-11001, Thermo Fisher Scientific, city, Waltham, Massachusetts, USA); anti-β-actin conjugated with horse radish peroxidase (#A3854, Sigma Aldrich, city, Munich, Germany).

### Immunoblotting

6 × 10^5^ cells were passaged on plates (10 cm in diameter), and allowed to attach overnight. Cells were treated with either DMSO (final concentration of 0.1%; control cells) or 30, 60, and 100 μM genistein for 48 h. Cells were lysed with a solution containing 1% Triton X-100, 0.5 mM EDTA, 150 mM NaCl, 50 mM Tris, pH 7.5, and a mixture of protease and phosphatase inhibitors (Roche Applied Science, Penzberg, Germany; #05892791001 and #11873580001), and cleared by centrifugation. Proteins were separated using the WES system (WES - Automated Western Blots with Simple Western; ProteinSimple, San Jose, California, USA), with 12–230 kDa Separation Module (#SM-W003), and detected with Anti-Mouse Detection Module (#DM-002), according to the manufacturer’s instruction. Staining with anti-β-actin antibody (1:25000) was used as an internal control to normalize the amounts of huntingtin.

### Fluorescence microscopy

4 × 10^4^ fibroblasts were passaged on coverslips in 12-well plates, and allowed to attach overnight. Cells were treated with either DMSO (control cells), or 30, 60, and 100 μM genistein for 48 h. The cells were fixed with 2% paraformaldehyde in phosphate buffered saline (PBS), and rinsed with 0.1% Triton X-100 in PBS. Then, the samples were blocked with 5% BSA and 1.5% glycine in PBS for 1 h. Following overnight incubation with primary antibody (1:1000) dissolved in PSB, the cells were rinsed with PBS 3 times, and incubated with secondary antibody dissolved in PBS (1:4000) for 2 h. After washing with PBS (5 times) coverslips were affixed to glass slides with a mounting medium, and next day, they were analyzed using the Nikon Eclipse E800 microscope.

### Statistical analysis

Data are presented as means ± SDs. Comparisons between two groups were performed by the Student’s *t* test, and comparison between several groups were tested by ANOVA. Statistical differences were considered significant at *p* < 0.05.

### Approval and accordance

All experimental protocols were approved by the Head of Department of Molecular Biology and Dean of Faculty of Biology, according to the procedures described in guidelines and regulations of the Vice-rector for Research of University of Gdańsk.

## Results

To investigate effects of genistein on patient-derived HD cells, we tested fibroblast cell lines obtained from four patients with confirmed HD diagnosis, and four volunteers without any HD symptoms and with no HD familial history as the control group. The experiments were performed with all cell lines, and each experiment was repeated at least 3 times. Then, results of each group (each set of 4 cell lines) were combined and analyzed.

In our previous studies (Pierzynowska et al. 2018b), we used HEK-293 cells transiently transfected with a plasmid encoding a fusion protein consisting of the 1st exon of *HTT* bearing 74 CAG repeats and the GFP protein. Therefore, when using anti-GFP antibodies, it was possible to specifically monitor levels of mHTT, without signals from endogenous HTT encoded by chromosomal *HTT*. In contrast, in patient-derived fibroblasts, both forms, i.e., HTT and mHTT, are present, because HD patients are heterozygotic for the *HTT* gene. The anti-huntingtin antibody detects both HTT and mHTT as the proteins differ only in the length of glutamine repeats. Thus, the major difference between control and HD cells should be reflected by the level of total HTT (HTT + mHTT), and the number of intracellular aggregates.

**Fig 1:**
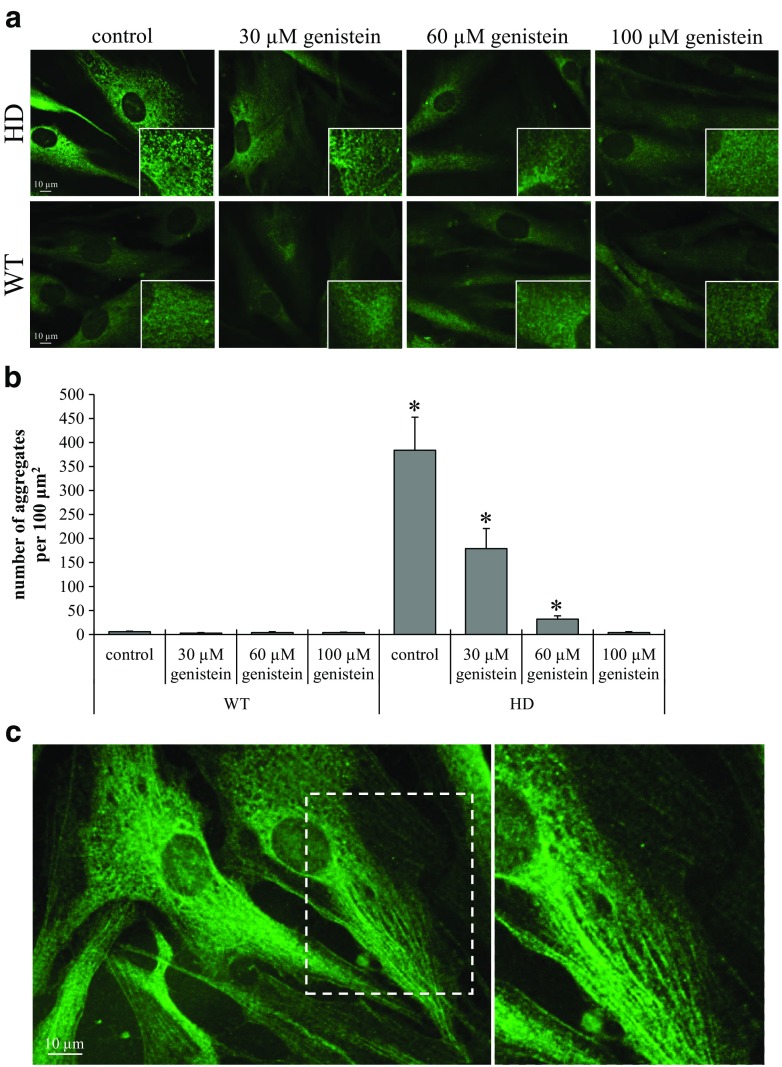
Genistein reduces the number of mHTT aggregates in human fibroblasts. Cultures of fibroblasts derived from four patients suffering from Huntington’s disease (HD) and four healthy persons (WT) were treated with either DMSO (control) or 30, 60 and 100 μM genistein, for 48 h. Cells were immuno-stained with anti-huntingtin antibody, secondary antibody conjugated with Alexa Fluor 488, and analyzed by fluorescent microscopy at 1000 x magnification. The number of aggregates per 100 μm^2^ were counted in 100 cells per culture. The experiments were performed in triplicates. Panel A demonstrates representative cells from each group. Panel B shows quantitative analysis of the number of aggregates, where bars represent mean values ± SD, and asterisks indicate statistically significant differences (*p* < 0.05) between WT and HD groups. Panel C demonstrates association of mHTT with cytoskeletal fibers in fibroblasts derived from HD patient. In panels A and C, white scale bars indicate 10 μm

As expected, when control fibroblasts were studied using fluorescent microscopy, HTT was uniformly distributed, and the fluorescent signals were moderate (Fig. 1). In contrast, HD fibroblasts revealed increased intensity of HTT staining that formed multiple aggregates apparently composed of mHTT. These aggregates tended to associate with the cytoskeletal fibers (Fig. 1).

The addition of genistein at different concentrations had no significant effect on control cells. However, in HD fibroblasts, HTT-specific signals were reduced significantly in a dose-dependent manner (Fig. 1). At the highest genistein concentration, the intensity of HTT signals approached the intensity seen in control fibroblasts.

**Fig 2:**
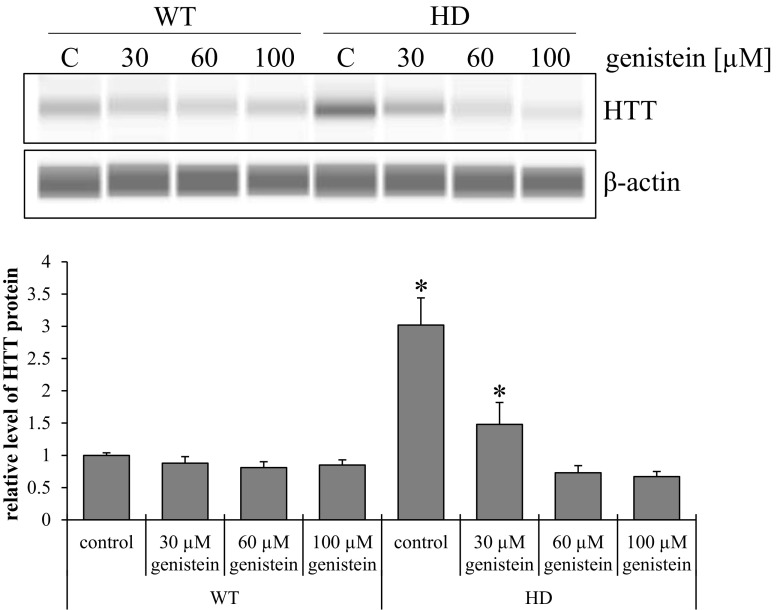
Genistein normalizes levels of HTT in HD fibroblasts. Cultures of fibroblasts derived from four HD patients (HD) and four healthy persons (WT) were treated with either DMSO (control) or 30, 60 and 100 μM genistein, for 48 h. Cellular proteins were separated and detected using the WES system. Four HD and four WT cell lines were used, and the analyses were performed in triplicates. Representative blots are shown in the upper panel, and quantitative analysis is presented in the lower panel. Bars represent mean values ± SD from three independent experiments. Asterisks indicate statistically significant differences (*p* < 0.05) between WT control and HD groups

Western-blot analysis revealed significantly higher levels of HTT in untreated HD relative to control cells (Fig. 2). The exposure of HD fibroblasts to genistein decreased cellular HTT levels, in the dose-dependent manner. At 60 and 100 μM genistein, HTT levels dropped to the levels in control fibroblasts.

## Discussion

The pathology of many neurodegenerative diseases feature the formation of protein aggregates (Aguzzi and O’Connor 2010). Therefore, one might suggest that enhanced degradation of protein aggregates would be the most effective way to treat these diseases. In fact, we (Pierzynowska et al. 2018a) and others (Guo et al. 2018) have proposed that autophagy, a major processes of protein degradation in cells, could be a promising target for therapeutic intervention to combat neurodegeneration.

HD can be considered a model for this group of neurodegenerative disorders (Morreale 2015). The expansion of CAG repeats in the *HTT* gene results in the generation of the pathogenic protein, mHTT, which accumulates and forms insoluble aggregates. Our recent studies showed that genistein, an efficient and safe stimulant of autophagy can correct HD phenotype in HEK-293 cells transfected with the *HTT* gene containing 74 CAG repeats (Pierzynowska et al. 2018b). This experimental system allowed for high level production of the mutant polypeptide and the formation of large amounts of intracellular aggregates, and thus, it was useful in testing efficiency of genistein action and in investigating the underlying mechanisms. However, the question remained whether genistein can effectively correct the HD phenotype of patient-derived cells. Therefore, we tested fibroblast lines derived from HD patients and controls. This model was chosen because HD fibroblast are unmodified cells derived directly from patients, and because they possess common features with HD neural cells (Sassone et al. 2009; Marchina et al. 2014; Petersen et al. 2014; Jędrak et al. 2017a).

Our present data clearly indicate that genistein is effective in normalizing HTT levels in HD fibroblasts as demonstrated by immunofluorescence and Western-blotting experiments. Because HTT levels were unchanged after treatment of control fibroblasts, we conclude that genistein induced specific degradation of mHTT, with no significant influence on normal HTT. This is in concordance with our previous studies in HEK-293 cells showing genistein-induced degradation of mHTT with 74 Q residues, but no degradation of mHTT with 23 Q residues (Pierzynowska et al. 2018b).

We observed that in HD fibroblasts, mHTT formed aggregates that associated with cytoskeletal fibers. Such results are compatible with recently reported observations that HTT can be associated with isoforms of α-actinin that bind actin filaments, and that mHTT localizes specifically to actin stress fibers (Tousley et al. 2019). Again, treatment with genistein resulted in disappearance of aggregates, and HTT distribution resembling the distribution in healthy cells.

In summary, our results demonstrate that genistein can correct HD phenotype in patient-derived cells. Of note, genistein might also be effective in other neurodegenerative diseases, e.g., Alzheimer’s disease (Pierzynowska et al. 2019), and Parkinson’s disease (Wu et al. 2018). Therefore, it is tempting to suggest that genistein, acting as an autophagy stimulator, can be considered a potential therapeutic agent for various neurodegenerative diseases that feature accumulation of misfolded proteins. Altogether, these results provide a rationale for further studies aimed at developing genistein-based therapy for HD and possibly other neurodegenerative disorders.
